# The Prevalence of Small Intestinal Bacterial Overgrowth in Patients with Non-Alcoholic Liver Diseases: NAFLD, NASH, Fibrosis, Cirrhosis—A Systematic Review, Meta-Analysis and Meta-Regression

**DOI:** 10.3390/nu14245261

**Published:** 2022-12-09

**Authors:** Anna Gudan, Dominika Jamioł-Milc, Victoria Hawryłkowicz, Karolina Skonieczna-Żydecka, Ewa Stachowska

**Affiliations:** 1 Department of Human Nutrition and Metabolomics, Pomeranian Medical University in Szczecin, ul. Władysława Broniewskiego 24, 71-460 Szczecin, Poland; 2 Department of Biochemical Sciences, Pomeranian Medical University in Szczecin, ul. Władysława Broniewskiego 24, 71-460 Szczecin, Poland

**Keywords:** microbiome, gut dysbiosis, small intestinal bacterial overgrowth, steatosis, NAFLD, NASH, cirrhosis

## Abstract

Bacterial overgrowth in the small intestine (SIBO) is a pathological growth of the intestinal microbiota in the small intestine that causes clinical symptoms and can lead to digestive and absorption disorders. There is increasing evidence that people with NAFLD have a distinct gut microflora profile as well metabolome changes compared to people without NAFLD. Thorough analysis of observational and RCT studies in the current databases (EMBASE, Web of Science, PubMed, Cinahl, Clinical Trials) was conducted from 3 November 2021 to 21 June 2022. The following inclusion criteria were applied: confirmed NAFLD, NASH, LIVER FIBROSIS, CIRRHOSIS due to steatosis; diagnostic methods of liver diseases—biopsy, elastography, transabdominal ultrasound; nonalcoholic fatty liver disease activity score; confirmed SIBO; diagnostic methods of SIBO–breath tests (hydrogen test; methane test and mix test; duodenal and jejunal aspiration before any type of intervention; adults above 18yo; number of participants ≥20; full articles. We excluded review articles, populations with HBV/HCV infection and alcohol etiology and interventions that may affect NAFLD or SIBO treatment. The quality of each study methodology was classified by means of the Cochrane Collaboration’s tool (RCT) and Newcastle—Ottawa Quality Assessment Scale adapted for cross-sectional, cohort and case-control studies. The random effects meta-analysis of outcomes for which ≥2 studies contributed data was conducted. The *I*^2^ index to measure heterogeneity and the χ^2^ test of homogeneity (statistically significant heterogeneity *p* < 0.05) were applied. For categorical outcome, the pooled event rate (effect size) was calculated. This systematic review was reported according to PRISMA reporting guidelines. We initially identified 6643 studies, from which 18 studies were included in final meta-analysis. The total number of patients was 1263. Accepted SIBO diagnostic methods were both available breath tests (n-total = 15) and aspirate culture (n-total = 3). We found that among patients with non-alcoholic liver diseases, the random overall event rate of SIBO was 0.350 (95% CI, 0.244–0.472), *p* = 0.017. The subgroup analysis regarding a type of diagnosis revealed that the lowest ER was among patients who developed simultaneously NAFLD, NASH and fibrosis: 0.197 (95% CI, 0.054–0.510) as compared to other annotated subgroups. The highest prevalence of SIBO was observed in the NASH subgroup: 0.411 (95% CI, 0.219–0.634). There were no statistically significant differences in the prevalence of SIBO in different subgroups (*p* = 0.854). Statistically significant heterogeneity between studies was estimated (*I*^2^ = 86.17%, *p* = 0.00). Egger’s test did not indicate a publication bias (*df* = 16, *p* = 0.885). A meta-regression using a random-effects model revealed that higher percentage of males in the population with liver diseases is a predisposing factor toward SIBO (Q = 4.11, *df* = 1, *p* = 0.0426 with coefficient = 0.0195, SE = 0.0096, Z = 2.03). We showed that the prevalence of SIBO in patients with chronic non-alcoholic liver diseases can be as high as 35%, and it increases with the percentage of men in the population. The prevalence of SIBO does not differ significantly depending on the type of chronic liver disease. Despite the high heterogeneity and moderate and low quality of included studies, our meta-analysis suggests the existence of a problem of SIBO in the population of patients with non-alcoholic liver diseases, and the presence of SIBO, in turn, determines the therapeutic treatment of such type of patients, which indicates the need for further research in this area. The study protocol was registered with the international Prospective Register of Systematic Reviews (PROSPERO ID: CRD42022341473).

## 1. Introduction

Bacterial overgrowth in the small intestine (SIBO) is a pathological growth of the intestinal microbiota in the small intestine that causes clinical symptoms and can lead to digestive and absorption disorders [1]. SIBO has been associated with disorders of the gut–brain axis, liver disorders, metabolic disorders, and impaired absorption of fats and nutrients [1,2,3]. The causes of SIBO include disorders of the gastrointestinal tract (e.g., in irritable bowel syndrome), anatomical abnormalities, postoperative adhesions, bypassing bariatric surgery, digestive disorders (e.g., in the course of pancreatitis), liver cirrhosis, old age, small intestine diverticula, poor diet, stress and medications [4]. The aftermath of SIBO includes bile salt deconjugation and impairment of fat digestion [5,6], fatty diarrhea, fat-soluble vitamin malabsorption, amino acid and disaccharide malabsorption, vitamin B12 deficiency, and progressive malnutrition and wasting [4]. SIBO causes excessive production of ammonia and increases the translocation of bacteria and the absorption of bacterial antigens into the bloodstream [7,8] and thus may promote the development of hepatic inflammation, steatosis and fibrosis [6].

Clinical symptoms of bacterial overgrowth in the small intestine are excessive accumulation and gas flow, abdominal distension, chronic diarrhea (watery or fatty), abdominal pain, abdominal fullness, constipation (when methanogens are overgrown in the intestine), weight loss and progressive malnutrition, edema (in the syndrome of protein loss from the gastrointestinal tract), symptoms of a deficiency of fat-soluble vitamins, erythema nodosum and maculopapular exanthema [4,6].The diagnosis of bacterial overgrowth in the small intestine is based on laboratory tests, respiratory tests, microbiological tests of aspirate, X-ray examinations of the gastrointestinal tract and endoscopy [1,9,10]. There is no single diagnostic test that allows for a clear diagnosis [10]. SIBO treatment includes the treatment of the underlying disease, the use of antibiotics (most often rifaximin, neomycin, metronidazole), nutritional treatment (some studies have shown a beneficial effect of the low FODMAPs diet on the reduction of symptoms of SIBO patients) [11], vitamin deficiency supplementation and supportive treatment (prokinetics, cholestryramine) [4]. Among the breathing tests for the diagnosis of bacterial overgrowth in the small intestine, there are two most common breathing tests: hydrogen and hydrogen–methane. A positive test result is an increase in exhaled hydrogen >20 ppm in the first 120 min of the test, an increase in methane >10 ppm throughout the test, the total increase in hydrogen and methane >15 ppm in the first 120 min [4]. Microbiological testing of intestinal aspirate from the small intestine is positive if the microbial content exceeds 103 CFU/mL [6].

Nonalcoholic fatty liver disease (NAFLD) is one of the most common chronic liver diseases worldwide [12]. NAFLD is defined by the spectrum of pathological changes occurring within the liver cells with the accumulation of lipids inside the hepatocytes. Disease progression includes non-alcoholic hepatitis (NASH), fibrosis, cirrhosis, and even hepatocellular carcinoma. NAFLD has been associated with metabolic disorders, insulin resistance, dyslipidemia, obesity, metabolic syndrome, cardiovascular disease, kidney disease, and inflammation [13,14]. The cause of fatty liver disease is still not fully understood. The challenge are patients with normal body weight and confirmed fatty liver. The cause of NAFLD progression in lean individuals is still unknown [15]. The current treatment for NAFLD in lean people is the reduction of visceral fat through a healthy diet and lifestyle, and the use of certain medications. Previous studies have shown a relationship between intestinal dysbiosis and the occurrence of fatty liver [16].

Intestinal dysbiosis is characterized by the growth of pathogenic bacteria and a decrease in the abundance and variety (richness) of commensal bacteria [16]. The mechanisms by which intestinal dysbiosis contributes to NAFLD include dysbiosis-induced disturbance of the intestinal barrier continuity, increased intestinal permeability, endotoxemia and lipopolysaccharide (LPS) accumulation, endogenous ethanol production, increased energy recovery from food or changes in choline and bile acid metabolism [17,18]. There is increasing evidence in this line of research that people with NAFLD have a distinct gut microflora profile as well metabolome changes compared to people with diagnosed NAFLD [19]. The altered microbiota produces a variety of hepatotoxic substances, including ammonia, indole, skatole, lipopolysaccharide, and pathogen-associated molecular patterns (PAMPs) [7,8,20]. The reaching of these substances to the liver and bacterial translocation may directly contribute to the formation and progression of NAFLD [20,21]. Disturbances of the intestinal microbiota are therefore present in patients with confirmed fatty liver, but further studies are still needed to determine whether NAFLD contributes to the development of intestinal dysbiosis or whether intestinal dysbiosis is one of the causes of NAFLD. The aim of this study was to assess the coexistence of small intestinal bacterial overgrowth (diagnosed by different breath tests or quantitive jejunal/duodenal aspiration) in adult men and women with non-alcoholic fatty liver disease (NAFLD), non-alcoholic hepatitis (NASH), liver fibrosis and cirrhosis proven by liver biopsy.

## 2. Materials and Methods

We performed a systematic review and a meta-analysis of observational studies and randomized controlled trials (RCTs) in accordance with the Preferred Reporting Items for Systematic Reviews and Meta-Analyses (PRISMA) guidelines [22]. The study protocol was registered with the international Prospective Register of Systematic Reviews (PROSPERO ID: CRD42022341473).

### 2.1. Search Strategy and Inclusion Criteria 

Three independent authors (A.G., D.J.M, V.H) searched Embase/PubMed/MEDLINE/Cinahl/Web of Science/Clinical Trials from database inception from 3 November 2021 till 21 June 2022 for observational studies and randomized controlled trials (RCTs) concerning prevalence, i.e., the frequency, of the occurrence of SIBO (small intestinal bacterial overgrowth) in the group of patients with selected non-alcoholic liver disorders (NAFLD, NASH, liver fibrosis, cirrhosis). The incidences of SIBO were not investigated. Only manuscripts in English were included. Searched databases and search string are presented in Table 1.

The following inclusion criteria were applied: confirmed NAFLD, NASH, LIVER FIBROSIS, CIRRHOSIS due to steatosis; diagnostic methods of liver diseases—biopsy, elastography, transabdominal ultrasound; nonalcoholic fatty liver disease activity score; confirmed SIBO; diagnostic methods of SIBO—breath tests (hydrogen test; methane test and mixed hydrogen-methane test); duodenal and jejunal aspiration before any type of intervention; adults above 18yo; number of participants ≥20 and full articles.

We excluded review articles, populations with HBV/HCV infection, alcohol etiology, children and interventions that may affect NAFLD or SIBO treatment, including herbal and pharmaceutical preparations (PPI, opioids), supplements, modified diet composition, reduction diet, physical activity, bariatric surgery, dyspepsia, IBS, intestinal dysmortility, small bowel diverticula, systemic sclerosis, abdominal surgery, coronary artery disease, diabetes, hypothyroism, Parkinson’s disease, rosacea, restless leg syndrome, etc.

Studies in hospital wards and specialist clinics were included. There was no geographic area restriction.

### 2.2. Study Selection Process

The study selection process was carried out in stages by three independent authors (A.G., D.J.M., V.H.). In the first step, the databases were screened in terms of compliance of the publication title with the assumptions. In the next step, the included publications were verified according to the abstract, and then the compliance of the full text with the inclusion criteria. Inconsistencies were resolved by the last author (E.S.), who acted as a clinical guarantor of the article.

### 2.3. Data Extraction

Data on sponsorship, blinding, setting, focus of the study, as well as patient characteristics (body mass index of patients diagnosed with non-alcoholic liver disease (NAFLD, NASH, fibrosis, cirrhosis) and SIBO assessed prior to any possible intervention; age and gender of patients diagnosed with non-alcoholic liver diseases and SIBO; liver enzyme levels in patients diagnosed with non-alcoholic liver diseases and SIBO assessed prior to any possible intervention; the stage of the disease assessed prior to any possible intervention,) and SIBO and non-alcoholic liver diseases diagnostic method used were independently extracted in accordance with the Preferred Reporting Items for Systematic Reviews and Meta-Analyses (PRISMA) [22] standard by three independent investigators (D.J.M., A.G., V.H.). Inconsistencies were resolved by the last author (E.S.), who acted as a clinical guarantor of the article.

### 2.4. Outcomes

Primary outcomes: prevalence (initial, before any intervention) of SIBO by type of the non-alcoholic liver diseases expressed as the number/percentage of patients diagnosed with SIBO.

For the calculation of the effect size (event rate), we abstracted the number of cases (patients with both NAFLD or cirrhosis and SIBO) among overall patients with liver diseases of our interests.

Secondary outcomes: prevalence of SIBO by gender, year of publication and SIBO diagnosis techniques among patients with the non-alcoholic liver diseases.

### 2.5. Data Synthesis and Statistical Analysis

The RCT and observational studies: cross-sectional, cohort, case-control and were included. Qualified studies were summarized in the text and relevant data presented in a tabular form. The random effects meta-analysis of outcomes for which ≥2 studies contributed data was conducted. The *I*^2^ index to measure heterogeneity and the χ^2^ test of homogeneity (statistically significant heterogeneity *p* < 0.05; considerable heterogeneity *I^2^* = 70% to 100%) were applied. The subgroup analysis by type of study and sensitivity analysis with a one-study-removed approach was used to estimate changes in heterogeneity values. For categorical outcome, the pooled event rate (effect size) was calculated. A subgroup analysis (regarding diagnosis) and random effects meta-regression analyses (regarding gender distribution in a population, testing method and year of publication) were conducted. Publication bias was assessed using a funnel plot and Egger’s test (*p* < 0.05). The Comprehensive Meta-Analysis software V3.3.070 (http://meta-analysis.com; Biostat Inc., Englewood, CO, USA) was used for calculations.

### 2.6. Risk of Bias

The quality of each study methodology was classified by two independent investigators (A.G. and D.J.M.) by means of the Cochrane Collaboration’s tool (RCT) [23], Newcastle—Ottawa Quality Assessment Scale adapted for cross-sectional, cohort and case-control studies [24]. Depending on the type of study, the following criteria were assessed: RCTs-selection, performance, detection, attrition, and reporting bias; case-control studies—selection, comparability, ascertainment of exposure; cross-sectional studies and cohort studies—selection, comparability, and assessment of outcome. According to RoB2 guidelines, we assumed that “Low risk of bias” determines the study judged to be at low risk of bias for all domains; “Some concerns”— the study is judged to raise some concerns in at least one domain for this result, but not to be at high risk of bias for any domain; “High risk of bias”—the study is judged to be at high risk of bias in at least one domain for this result or the study is judged to have “some concerns” for multiple domains in a way that substantially lowers confidence in the result. The maximum star rating was 9 for cohort studies and case-control studies and 10 for cross-sectional studies. In the case of cohort studies and case-control studies, less than 5 stars indicated low quality, 5–7 stars indicated moderate quality, and 8–9 stars indicated high quality. On the other hand, in cross-sectional studies, less than 6 stars were considered low quality, 6–8 moderate quality and 9–10 high quality.

## 3. Results

### 3.1. Search Results

**Figure 1:**
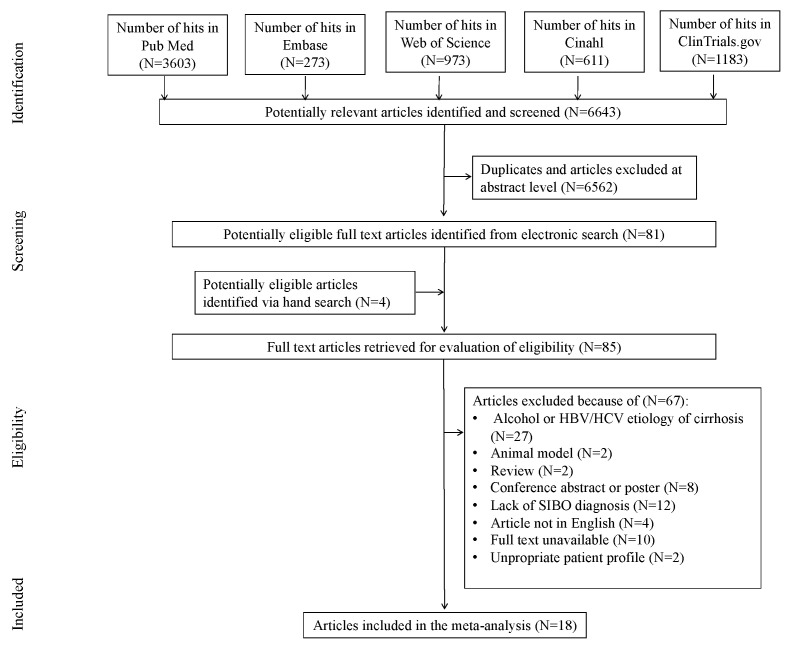
Preferred Reporting Items for Systematic Reviews and Meta-Analyses (PRISMA) study flowchart depicting search strategy and study selection.

The initial search yielded 6643 hits. A total of 6562 studies were excluded, being duplicates and/or after evaluation on the title/abstract level. There were 4 additional articles identified via hand search. Overall, 81 full-text articles were incorporated into the final abstraction level. Of those, 67 were excluded due to not fitting inclusion criteria: the reasons for exclusion were alcohol or HBV/HCV etiology (N = 27), animal model (N = 2), review (N = 2), conference abstract or poster (N = 8), lack of SIBO diagnosis (N = 12), article not in English (N = 4), full text unavailable (N = 10) or inappropriate patient profile (N = 2) (Figure 1). This yielded 18 studies that were included in the meta-analysis.

### 3.2. Study, Patient and Treatment Characteristics

The total number of 18 observational studies included in our meta-analysis were conducted in 14 different countries. We finally analysed one cohort study [25], cross-sectional studies (n = 5) [26,27,28,29,30], case-control studies (n = 8) [31,32,33,34,35,36,37,38] and randomized controlled trials (n = 4) [39,40,41,42]—Table 2. The total number of patients from all studies was 1263. This number refers to the total number of patients finally analysed from all subgroups (NAFLD, NASH, fibrosis, cirrhosis). The mean percentage of males was 40.14. The range of age of studies’ participantswas 20–78 years. The liver diseases were diagnosed by liver biopsy and Transient Elastography ultrasound. SIBO was diagnosed with 14C-D-Xylose and Lactulose Breath Test (n = 1) [38], Glucose Hydrogen Breath Test (n = 8) [27,28,30,32,34,35,42,43], Lactose Hydrogen Breath Test (n = 1) [25], Lactulose Hydrogen Breath Test (n = 3) [36,37,41], Lactulose Hydrogen–Methane Breath Test (n = 3) [26,31,40], Quantitative Duodenal Aspirate Culture (n = 2) [29,33] and Quantitative Jejunal Aspirate Culture (n = 1) [32]. Two tests were run simultaneously in one study: Quantitative Jejunal Aspirate Culture and Glucose Hydrogen Breath Test—Table 3.

### 3.3. The Quality of Studies

None of the four clinical trials [39,40,41,42] achieved an overall “Low risk of bias” rating across all domains evaluated. All studies in two domains were rated “some concerns” and in at least one domain “High risk of bias” (Supplementary Table S1). Therefore, all RCT studies received an overall “High risk of bias” rating. The only cohort study [25] was rated seven stars (moderate quality)—Supplementary Table S2. Among case-control studies (n = 8) [31,32,33,34,35,36,37,38], the lowest rating was three stars—low quality (two studies) [33,37]—and the highest was eight stars (high quality, one study) [34]. Overall, three studies [31,33,37] were low quality. Four studies [32,35,36,38] were rated as moderate quality—Supplementary Table S3. On the other hand, in the cross-sectional studies group (n = 5) [26,27,28,29,30], one study [26] received 5 stars (the lowest rating, low quality) and one study [30] received 10 stars (the maximum rating, high quality). The quality of three studies [27,28,29] was moderate-level—Supplementary Table S4.

### 3.4. Prevalence of SIBO and Random Effects Meta-Regression Analyses

**Figure 2:**
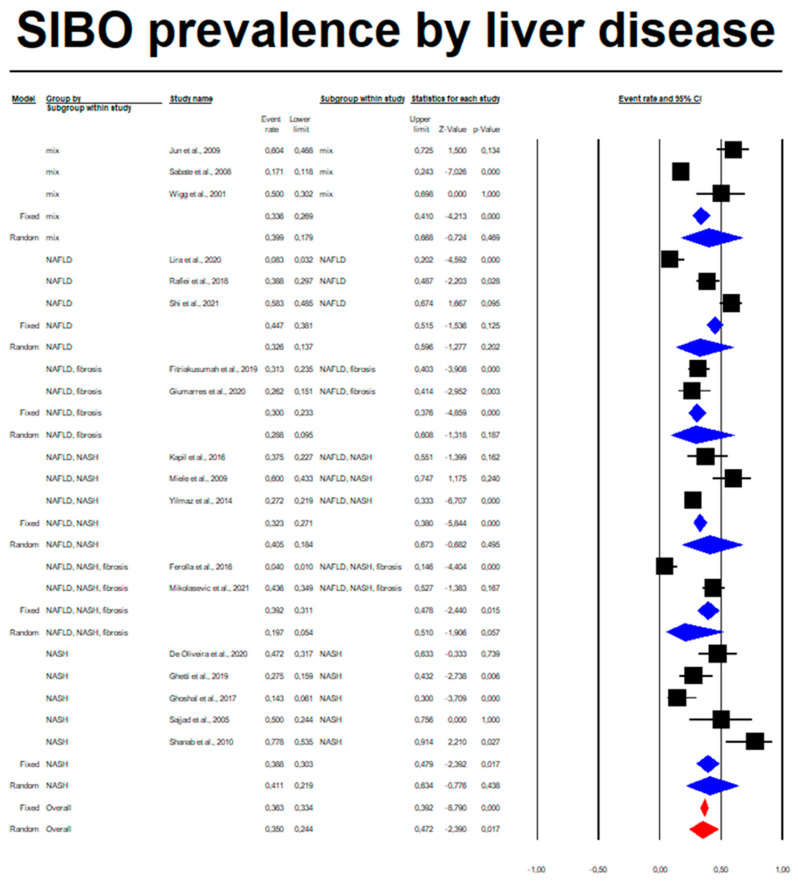
SIBO rate regarding types of liver diseases: Mix (NASH, cirrhosis, fibrosis) Q = 33.867, *df*(Q) = 2, *p* = 0.00, *I*-squared = 94.1; NAFLD Q = 26.358, *df*(Q) = 2, *p* = 0.00, *I*-squared = 92.412; NAFLD, fibrosis Q = 0.383, df(Q) = 1, *p* = 0.536, *I*-squared = 0; NAFLD, NASH Q = 14.60, *df*(Q) = 2, *p* = 0.001, *I*-squared = 85.876; NAFLD, NASH, fibrosis Q = 15.349, *df*(Q) = 1, *p* = 0.00, *I*-squared = 93.485; NASH Q = 20.523, *df*(Q) = 4, *p* = 0.00, *I*-squared = 80.510) [25,26,27,28,29,30,31,32,33,34,35,36,37,38,39,40,41,42].

**Figure 3:**
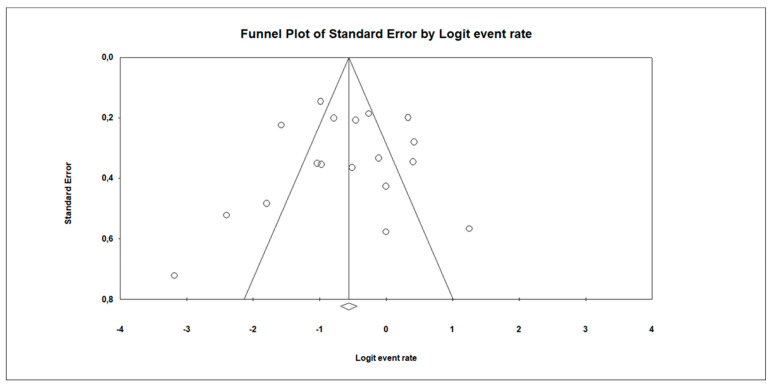
Funnel plot for event rate in the present meta-analysis. Egger’s test: *p* = 0.885.

Among patients with non-alcoholic liver diseases, the random overall event rate of SIBO was 0.350 (95% CI, 0.244–0.472), *p* = 0.017 (Figure 2 and Table 4). The subgroup analysis regarding a type of diagnosis revealed that the lowest ER was among patients who developed simultaneously NAFLD, NASH and fibrosis: 0.197 (95% CI, 0.054–0.510) as compared to other annotated subgroups (Table 4), whereas the highest prevalence of SIBO was observed in the NASH subgroup: 0.411 (95% CI, 0.219–0.634). Overall, however, there were no statistically significant differences in the prevalence of SIBO in different subgroups (*p* = 0.854). Statistically significant heterogeneity between studies was estimated (*I*^2^ = 86.17%, *p* = 0.00). The analysis regarding type of study by means of meta-regression was also performed. We did not observe any significant association (coefficient: −0.7155, SE: 0.4714; *p* = 0.1290). Doing a subgroup analysis by type of study, we found that *I*^2^ in observational studies (n = 14) was 86.646% and, in cases of RCT (n = 4), 75.113%; *p* = 0.120. Moreover, a sensitivity analysis with a one-study-removed approach did not change the *I*^2^ (86.17%). Egger’s test did not indicate a publication bias (*df* = 16, *p* = 0.885) (Figure 3).

**Figure 4:**
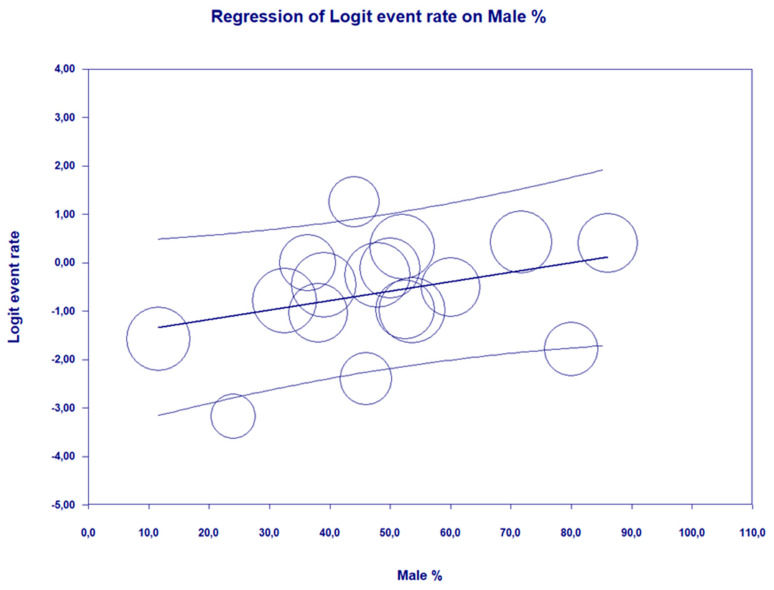
The meta-regression on log risk ratio of SIBO prevalence depending on percentage of men in population with liver diseases (coefficient = 0.0195, *p* = 0.0426).

A meta-regression using a random effects model revealed that higher percentage of males in the population with liver diseases is a predisposing factor toward SIBO (Q = 4.11, *df* = 1, *p* = 0.0426 with coefficient = 0.0195, SE = 0.0096, Z = 2.03) (Table 5 and Figure 4). The meta-regression analysis showed no relationship between the date of publication of the study or the type of diagnostic test used and the prevalence of SIBO (Figures S1 and S2).

## 4. Discussion

### 4.1. Principal Findings

In recent times, scientific research indicates a possible role of small intestinal bacterial overgrowth in both the formation and progression of liver diseases (NAFLD, NASH, fibrosis, cirrhosis) [27,44,45]. The entire area of the intestine is anatomically connected to the liver via a portal vein [7]. The theory of autointoxication proposed in the last century by Llewellyn J. et al. assumes that the portal vein pathway, from the intestine to the liver, reaches toxic substances that change the functions and metabolism of the liver itself [8,34]. This mechanism of endotoxemia has been confirmed in new studies to date [8,46,47]. Disturbances in the enterohepatic axis, including disturbances in the composition and function of the gut microbiome seem to play a role in the incidence and progression of chronic liver disease [7,18]. Quantitative and qualitative disturbances in the intestinal microbiome, including the presence of SIBO, contribute to a greater accumulation of Gram-negative bacteria [32]. A common feature of many Gram-negative bacteria is the presence of lipopolysaccharide (LPS), which causes inflammation in situ, disrupts the integrity of the intestinal barrier and penetrates into the host organism [45]. It has been shown that the influx of LPS into the liver contributes to the progression of steatosis and liver fibrosis [45,48]. Moreover, in patients with cirrhosis of the liver and impaired detoxification function of this organ, LPS may contribute to the development of hepatic encephalopathy [49]. Additionally, disturbance of the intestinal microbiome can lead to endogenous ethanol production [50], thus directly predisposing the patient to NAFLD by increasing oxidative stress and triglyceride accumulation directly in the liver [18]. Another mechanism by which untreated SIBO worsens the prognosis of patients with liver disease is the formation of nutritional deficiencies. Excessive bacterial growth causes malabsorption of valuable lipotropic components, including choline and vitamin B12 [6,51]. Deficiency of these nutrients has been shown to affect the progression of fatty liver and increase the risk of NAFLD formation [50].

In our meta-analysis, we showed that the prevalence of SIBO increases with the percentage of men in the population of people with chronic and non-alcoholic liver disease. The available literature indicates several potential mechanisms of this phenomenon. The role of sexual dimorphism in the development and progression of the disease is mainly based on the protective role of estrogens in premenopausal women [52]. Studies on animal models as well as studies on humans indicate a different metabolism of fat in women than in men [52,53]. Premenopausal women have a greater ability to metabolize fatty acids towards ketone bodies rather than towards very-low-density lipoproteins (vLDL) [52]. Moreover, in a mouse model, it was shown that female mice showed greater browning of white adipose tissue, which contributed to a significant improvement in insulin sensitivity and was protective against experimental NAFLD associated with methionine and choline deficiencies in the diet [54,55,56].

### 4.2. Results in the Context of Other Meta-Analyses

To date, studies have shown a link between the prevalence of SIBO and more severe liver disease [32,44,45,49,57,58,59]. Moreover, patients with liver disease who had minimal hepatic encephalopathy also had a higher prevalence of SIBO compared to patients with hepatic disease without minimal hepatic encephalopathy [49,60]. We hypothesize that undiagnosed and untreated SIBO can significantly worsen the course of the disease and delay recovery, especially when the disease is still in a reversible stage (e.g., NAFLD). In our meta-analysis we found that up to 35% of patients with nonalcoholic liver disease may suffer from SIBO. This is over one-third of the entire population of patients in whom the effectiveness of the primary disease treatment may be significantly impaired by the presence of SIBO. A meta-analysis conducted in 2017 by Shah A. et al. [61] is largely consistent with the results obtained in our study (35.80% for breath test and 68.31% for culture technics). There was a difference in the SIBO event rate depending on the diagnostic method used. The higher percentage of SIBO recorded when examining aspirates is probably due to the fact that it is the most accurate method of SIBO diagnostics, described as the gold standard [9,10]. However, the results of other studies do not unequivocally confirm this relationship. As in our meta-analysis, Shah A. et al. also showed that there is no significant difference in the population of liver patients between different diseases (here: NAFLD, NASH, fibrosis, cirrhosis) [61]. In 2020, Wijarnpreecha K. et al. [62] conducted a meta-analysis in which they assessed the incidence of SIBO in the NAFLD patient population. This study demonstrated a significant association between NAFLD and SIBO with the pooled odds ratio of 3.82 (95% confidence interval, 1.93–7.59; *I*^2^ 65%) [62]. The above two meta-analyses are the only works dealing with this topic so far.

In our study, we showed that the prevalence of SIBO increases with the percentage of men in the population of people with chronic liver disease of non-alcoholic origin. The studies conducted so far confirm this relationship [63,64,65]. It has been shown that men are statistically more likely to suffer from liver diseases than women in the premenopausal age [52]. The team of Riazi K. et al. [53] showed that prevalence of NAFLD was significantly higher in men than in women (39.7% [36.6–42.8] vs. 25.6% [22.3–28.8]; *p* < 0.0001). Considerable heterogeneity between studies of both NAFLD prevalence (*I*^2^ = 99.9%) and NAFLD incidence (*I*^2^ = 99.9%) was observed [53]. On the other hand, the frequency of SIBO in women vs men is not exactly known. It is known that among IBS patients, women are more likely to have severe symptoms and coexistent anxiety or depression [66].

### 4.3. Strengths of the Meta-Analysis

This is the first meta-analysis performed in 2022 to analyze the prevalence of SIBO in the population of patients suffering from non-alcoholic chronic liver diseases. The advantage of our meta-analysis is taking into account various diagnostic methods of SIBO, as well as taking into account many different chronic liver diseases both pooled and by subgroups.

### 4.4. Limitations of the Meta-Analysis

Our study included various SIBO diagnostic methods and different diagnostic devices; thus, it was impossible to avoid differences in technical factors, e.g., calibration of the device itself. Moreover, different diagnostic methods show different sensitivity; hence, the actual event rate may differ from the results presented so far. In our meta-analysis, statistically significant heterogeneity between studies was estimated (*I*^2^ = 86.17%, *p* = 0.00) and the quality of the studies were low or moderate (8 studies rated as high risk of bias, 9 studies rated as moderate and 1 study of high quality out of 18). Different age and gender distributions were taken into account.

## 5. Conclusions

Summing up, our meta-analysis showed that the prevalence of SIBO in patients with chronic nonalcoholic liver disease can be as high as 35% of the total population, and the prevalence increases with the percentage of men in the population. The prevalence of SIBO does not differ significantly depending on the type of chronic liver disease. Due to the significant heterogeneity and quality of the studies (out of 18, 8 studies rated as high risk of bias, 9 studies rated as moderate and 1 study rated as high quality), the result should be treated with caution. Despite the above, our meta-analysis suggests the existence of a problem of SIBO in the population of patients with non-alcoholic liver diseases, which indicates the need for further research in this area.

We also draw attention to the problem of the lack of diagnosis of SIBO in patients with liver diseases. It should be borne in mind that the treatment of a patient diagnosed both with NAFLD and SIBO is different and requires additional clinical (as well as antibiotic therapy) and dietary management.

## Figures and Tables

**Table 1:** Searched databases and search string.

Database	Search String
Embase	((‘adult’/exp OR ‘adult’ OR ‘adults’ OR ‘grown-ups’ OR ‘grownup’ OR ‘grownups’) AND (‘nonalcoholic fatty liver’/exp OR ‘nafld (nonalcoholic fatty liver disease)’ OR ‘non-alcoholic fatty liver disease’ OR ‘non-alcoholic hepato-steatosis’ OR ‘non-alcoholic hepatosteatosis’ OR ‘non-alcoholic liver steatosis’ OR ‘non-alcoholic steatotic hepatopathy’ OR ‘non-alcoholic fld’ OR ‘non-alcoholic fatty liver’ OR ‘non-alcoholic fatty liver disease’ OR ‘non-alcoholic hepatic steatosis’ OR ‘nonalcoholic fld’ OR ‘nonalcoholic fatty liver’ OR ‘nonalcoholic fatty liver disease’ OR ‘nonalcoholic hepatic steatosis’ OR ‘nonalcoholic hepatosteatosis’ OR ‘nonalcoholic liver steatosis’) OR ‘nonalcoholic steatohepatitis’/exp OR ‘nash (nonalcoholic steatohepatitis)’ OR ‘non-alcohol steato-hepatitis’ OR ‘non-alcohol steatohepatitis’ OR ‘non-alcoholic steato-hepatitis’ OR ‘non-alcohol steato-hepatitis’ OR ‘non-alcohol steatohepatitis’ OR ‘non-alcoholic steatohepatitis’ OR ‘non-alcoholic steatosis hepatitis’ OR ‘non-alcoholic steatotic hepatitis’ OR ‘nonalcohol steato-hepatitis’ OR ‘nonalcohol steatohepatitis’ OR ‘nonalcoholic fatty liver inflammation’ OR ‘nonalcoholic steato-hepatitis’ OR ‘nonalcoholic steatohepatitis’ OR ‘nonalcoholic steatosis hepatitis’ OR ‘nonalcoholic steatotic hepatitis’ OR ‘liver fibrosis’/exp OR ‘fibrosis, liver’ OR ‘fibrous hepatic disease’ OR ‘hepatic fibrosis’ OR ‘liver fibrosis’ OR ‘liver periportal fibrosis’ OR ‘periportal fibrosis’ OR ‘liver cirrhosis’/exp OR ‘cirrhosis’ OR ‘cirrhosis hepatis’ OR ‘cirrhosis, liver’ OR ‘cryptogenic liver cirrhosis’ OR ‘dietary cirrhosis’ OR ‘dietary liver cirrhosis’ OR ‘hepatic cirrhosis’ OR ‘liver cirrhosis’ OR ‘postnecrotic liver cirrhosis’) AND (‘small intestinal bacterial overgrowth’/exp OR ‘sbbo (small bowel bacterial overgrowth)’ OR ‘sibo’ OR ‘sibo syndrome’ OR ‘bacterial overgrowth syndrome (small intestine)’ OR ‘contaminated small bowel syndrome’ OR ‘enteral bacterial overgrowth’ OR ‘enteric bacteria overgrowth’ OR ‘enteric bacterial overgrowth’ OR ‘small bowel bacteria overgrowth’ OR ‘small bowel bacterial over growth’ OR ‘small bowel bacterial overgrowth’ OR ‘small bowel bacterial overgrowth syndrome’ OR ‘small bowel intestinal overgrowth’ OR ‘small gut bacterial overgrowth’ OR ‘small intestinal bacteria overgrowth’ OR ‘small intestinal bacterial over-growth’ OR ‘small intestinal bacterial overgrowth’ OR ‘small intestinal bacterial overgrowth syndrome’ OR ‘small intestinal bowel overgrowth’ OR ‘small intestinal overgrowth’ OR ‘small intestine bacteria overgrowth’ OR ‘small intestine bacterial over-growth’ OR ‘small intestine bacterial overgrowth’ OR ‘small intestine bacterium overgrowth’ OR ‘small intestine overgrowth’ OR ‘upper gut bacterial overgrowth’)
PubMed/Cinahl/Web of Science	(SIBO OR small intestinal bacterial overgrowth OR breath test* OR intestinal microbiology) AND (NAFLD OR “non-alcoholic fatty liver” OR NASH OR liver fibrosis OR cirrhosis OR steatohepatitis)
ClinTrials.Gov	NAFLD OR NASH OR hepatic fibrosis OR cirrhosis

**Table 2:** Studies characteristics.

	Study Description		Number of Patients with Liver Diseases		Sample Characteristics			
No	Overall Study Characteristics (First Author, Year, Country)	Type of the Study	N Randomized	N Analyzed	Type of Liver Disease	BMI Overall	Age (Years) Mean ± SD Median (Range)	Male (%)
1	De Oliveira J.M. et al., 2020, Brazil [26]	cross-sectional	45	36	NASH	ND	48.38 ± 10.24	50
2	Ferolla S.M. et al., 2016, Brazil [39]	RCT	50	50	NAFLD, NASH, fibrosis	>30 kg/m^2^	57.3 (25–74)	24
3	Fitriakusumah, Y et al., 2019, Indonesia [27]	cross-sectional	ND	160	NAFLD, fibrosis	>25 h)	58 (22–78) ^(a)^	32.5 ^(b)^
4	Ghetti, F.D.F et al., 2019, Brazil [40]	open label clinical trial	44	40	NASH	ND	49.45 ± 2.4	52.5
5	Ghoshal, U.C. et al., 2017, India [32]	case-control	38	35	NASH	25.4 (20.4–37.5) ^(e)^	37 (20–54)	80
6	Guimares V.M. et al., 2020, Brazil [41]	open label clinical trial	42	42	NAFLD, fibrosis	31.7 ± 0.8	55.5 ± 1.75	38.1
7	Jun D. et al., 2009, Korea [31]	case-control	53	53	cirrhosis	ND	55.1 ± 10.6	71.7
8	Kapil S. et al., 2016, India [33]	case-control	60	32	NAFLD, NASH	27.3 ± 4.3	38.7 ± 10.4	60
9	Lira M.M.P. et al., 2020, Brazil [28]	cross-sectional	48	48	NAFLD	29.3 (26.7–31.9) ^(c)^ 35.2 (31.4–39.0) ^(d)^	43.1 (38.3–47.9) ^(c)^; 53.3 (49.1–57.5) ^(d)^	46
10	Miele L. et al., 2009, Italy [34]	case-control	35	35	NAFLD, NASH	26.19	42 (32–54)	86
11	Mikolasevic I. et al., 2021, Croatia [29]	cross-sectional	117	117	NAFLD, NASH, fibrosis	33.4	58.3 ± 11.7	47.9
12	Rafiei R. et al., 2018, Iran [30]	cross-sectional	98	98	NAFLD	ND	48.5 ± 12.1	39
13	Sabaté J.M. et al., 2008, France [35]	case-control	146	127	severe steatosis, fibrosis, NASH	>40	40.7 ± 11.4	11.6
14	Sajjad A. et al., 2005, United Kingdom [42]	RCT	12	12	NASH	32	54 (35–69)	ND
15	Shanab A.A. et al., 2010, Ireland [36]	case-control	18	18	NASH	30	51.17 ± 2.4	44
16	Shi H. et al., 2021, China [37]	case-control	103	103	NAFLD	ND	48.52 ± 12.34	52
17	Wigg A.J. et al., 2001, Australia [38]	case-control	22	22	NASH, fibrosis	30	54 ± 17	36.36
18	Yilmaz Y. et al., 2014, Turkey [25]	cohort	235	235	NAFLD, NASH	ND	ND	53.6

^(a)^ age refers to the entire study group, i.e., patients with NAFLD (71.9%) and other metabolic diseases (28.1%); ^(b)^ number of male refers to the entire study group, i.e., patients with NAFLD (71.9%) and other metabolic diseases (28.1%); ^(c)^ for NAFLD LRAF group; ^(d)^ for NAFLD—HRAF group; ^(e)^ a total of twenty of 35 (57.1%) patients with NASH had BMI > 25 kg/m^2^, which has been defined as obesity in India; N = the number of all patients in the whole study, i.e., the group (with liver disease) in which the prevalence of SIBO was studied, i.e., the sum of SIBO patients and without SIBO; ND—no data.

**Table 3:** Studies characteristics.

	Study Description	SIBO Prevalence			Comorbidities	
No	Overall Study Characteristics (First Author, Year, Country)	SIBO (n)	N	Method of SIBO Diagnosis	Comorbidities (%)	Type of Comorbidities
1	De Oliveira J.M. et al., 2020, Brazil [26]	17	36	Lactulose Hydrogen–Methane Breath Test	58.3-obesity; 25-high glucose; 69.4 -high TG; 41.7-SAH	obesity, high glucose, high TG, SAH, MS
2	Ferolla S.M. et al., 2016, Brazil [39]	2	50	Glucose Hydrogen Breath Test	98	obesity, T2D, metabolic syndrome, SAH
3	Fitriakusumah, Y et al., 2019, Indonesia [27]	36	115	Glucose Hydrogen Breath Test	94.8	T2D, dyslipidemia, obesity- BMI > 25 (Asia Pacific criteria), MS, central obesity- WHO criteria for Asian population
4	Ghetti, F.D.F et al., 2019, Brazil [40]	11	40	Lactulose Hydrogen–Methane Breath Test	42.5-SAH; 20-T2D	T2D; SAH;
5	Ghoshal, U.C. et al., 2017, India [32]	5	35	Quantitative Jejunal Aspirate Culture; Glucose Hydrogen Breath Test	65.7	obesity ^(a)^, T2D
6	Guimares V.M. et al., 2020, Brazil [41]	11	42	Lactulose Hydrogen Breath Test	73.4-MS; 52.3-T2D	MS, T2D
7	Jun D. et al., 2009, Korea [31]	32	53	Lactulose Hydrogen–Methane Breath Test	ND	ND
8	Kapil S. et al., 2016, India [33]	12	32	Quantitative Duodenal Aspirate Culture	75	insulin resistance, overweight, obesity, central obesity, MS
9	Lira M.M.P. et al., 2020, Brazil [28]	4	48	Glucose Hydrogen Breath Test	ND	T2D, dyslipidemia, hypertension
10	Miele L. et al., 2009, Italy [34]	21	35	Glucose Hydrogen Breath Test	ND	MS
11	Mikolasevic I. et al., 2021, Croatia [29]	51	117	Quantitative Duodenal Aspirate Culture	44.4-T2D; 75.2-SAH; 75.3-dyslipidemia; 73.5-MS	T2D, SAH, dyslipidemia, MS
12	Rafiei R. et al., 2018, Iran [30]	38	98	Glucose Hydrogen Breath Test	51	MS
13	Sabaté J.M. et al., 2008, France [35]	24	140	Glucose Hydrogen Breath Test	100	morbid obesity, sleep apnoea, T2D, cardiovascular disease, SAH, dyslipidaemia, MS
14	Sajjad A. et al., 2005, United Kingdom [42]	6	12	Glucose Hydrogen Breath Test	42	T2D
15	Shanab A.A. et al., 2010, Ireland [36]	14	18	Lactulose Hydrogen Breath Test	ND	T2D, gall stones, depression, fatigue
16	Shi H. et al., 2021, China [37]	60	103	Lactulose Hydrogen Breath Test	ND	ND
17	Wigg A.J. et al., 2001, Australia [38]	11	22	14C-D-Xylose and Lactulose Breath Tests	40.9 ^(b)^	T2D, glucose intolerance, hyperlipidemia
18	Yilmaz Y. et al., 2014, Turkey [25]	64	235	Lactose Hydrogen Breath Test	71.4 ^(b)^	T2D, MS

^(a)^ >25 kg/m^2^, which has been defined as obesity in India; ^(b)^ one person may have had several diseases. N = the number of all patients in the whole study, i.e., the group (with liver disease) in which the prevalence of SIBO was studied, i.e., the sum of SIBO patients and without SIBO; n—number of SIBO patients in a given group with the number N; ND—no data; SAH—systolic arterial hypertension; T2D—type 2 diabetes; MS—metabolic syndrome; TG—triglycerides.

**Table 4:** Effect size, confidence interval and heterogeneity in study subgroups and overall.

		Effect Size and 95% Cl				Heterogeneity			
Subgroup	Studies (N)	Event Rate	Lower Limit	Upper Limit	*p*	Q	*df*(Q)	*p*	*I* ^2^
Mix	3	0.399	0.179	0.668	0.469	33.867	2	0.000	94.094
NAFLD	3	0.326	0.137	0.596	0.202	26.358	2	0.000	92.412
NAFLD, fibrosis	2	0.288	0.095	0.608	0.187	0.383	1	0.536	0.000
NAFLD, NASH	3	0.405	0.184	0.673	0.495	14.160	2	0.001	85.876
NAFLD, NASH, fibrosis	2	0.197	0.054	0.510	0.057	15.349	1	0.000	93.485
NASH	5	0.411	0.219	0.634	0.438	20.523	4	0.000	80.510
Overall	18	0.350	0.244	0.472	0.017	122.925	17	0.000	86.170

**Table 5:** Meta-regression analysis for % of males, type of testing method and year of publication on the SIBO prevalence.

Covariates	Number of Studies	Meta-Regression	
		Coefficient	*p*
% Male	17	0.0195	0.0426
Testing method:	18		0.2993
Glucose		−1.0196	
Lactose		−0.9828	
Lactulose		0.1078	
Quantitive Jejunal Aspiration		−0.3746	
Quantitive Duodenal Aspiration		−1.7918	
Year of publication	18	−0.0450	0.1675

## Supplementary Materials

The following supporting information can be downloaded at: https://www.mdpi.com/article/10.3390/nu14245261/s1, Figure S1: The meta-regression on log risk ratio of SIBO prevalence depending on year of publication (coefficient = −0.0450, *p* = 0.1675); Figure S2: The meta-regression on log risk ratio of SIBO prevalence depending on testing method (*p* = 0.2993); Table S1: The risk of bias of RCTs by means of the Cochrane Collaboration’s tool; Table S2: Newcastle–Ottawa Scale scoring for cohort studies; Table S3: Newcastle–Ottawa Scale scoring for case–control studies; Table S4: Newcastle–Ottawa Scale scoring for cross–sectional studies.

## Abbreviations

14CDXLBT	14C-D-Xylose and Lactulose Breath Tests
FODMAPs	Fructose, Oligosaccharides, Disaccharides, Monosaccharides and Polyols
GHBT	Glucose Hydrogen Breath Test
HBV	Hepatitis virus B
HCV	Hepatitis virus C
IBS	Irritable Bowel Syndrome
LCHBT	Lactose Hydrogen Breath Test
LHBT	Lactulose Hydrogen Breath Test
LHMBT	Lactulose Hydrogen-Methane Breath Test
NAFLD	Non-alcoholic Fatty Liver Disease
NASH	non-alcoholic hepatitis
PPIs	Proton Pump Inhibitors
PRISMA	Preferred Reporting Items for Systematic Reviews and Meta-Analyses
QDAC	Quantitative Duodenal Aspirate Culture
QJAC	Quantitative Jejunal Aspirate Culture
RCT	Randomized Controlled Trial
ROB	Risk of Bias
SIBO	Small Intestinal Bacterial Overgrowth
