# Supplementary material for: The Prevalence of Small Intestinal Bacterial Overgrowth in Patients with Non-Alcoholic Liver Diseases: NAFLD, NASH, Fibrosis, Cirrhosis—A Systematic Review, Meta-Analysis and Meta-Regression
Source: Nutrients. 2022 Dec 9;14(24):5261. doi: 10.3390/nu14245261 (PMC9783356; doi:10.3390/nu14245261)
Supplement: Supplementary file 1 [file nutrients-14-05261-s001.zip › nutrients-2029262-supplementary.pdf]

**Table S1.** The risk o bias of RCTs by means of the Cochrane Collaboration's tool.

| Author, Year | Sequence generation | Allocation concealment | Blinding of participants and personnel | Blinding of outcome assessment | Incomplete outcome data addressed | Selective reporting | Other bias | Overall risk-of-bias |
|---|---|---|---|---|---|---|---|---|
| Ferolla, S.M; 2016 | SC | H | L | L | L | L | SC | H |
| Sajjad, A.; 2005 | SC | SC | H | H | L | L | L | H |
| Ghetti, F.D.F.; 2019 | L | L | SC | H | L | L | SC | H |
| Guimares, V.M.; 2020 | H | SC | H | H | L | L | SC | H |

H - High risk of bias; L- Low risk of bias; SC - Some concerns

**Table S2.** Newcastle–Ottawa Scale scoring for cohort studies.

| Author, Year | Summary: Selection bias (max. four stars) | Summary: Comparability (max. two stars) | Summary: Outcome (max. three stars) | Total No. Stars (Max. nine stars) |
|---|---|---|---|---|
| Yilmaz, Y; 2014 | 4 | 2 | 1 | 7 |

**Table S3.** Newcastle–Ottawa Scale scoring for case–control studies.

| Author, Year | Summary: Selection bias (max. four stars) | Summary: Comparability (max. two stars) | Summary: Exposure (max. three stars) | Total No. Stars (Max. nine stars) |
|---|---|---|---|---|
| Ghoshal U.C.; 2017 | 3 | 0 | 2 | 5 |
| Jun D.W.; 2010 | 2 | 0 | 2 | 4 |
| Kapil S.; 2016 | 2 | 0 | 1 | 3 |
| Miele L.; 2009 | 4 | 1 | 3 | 8 |
| Sabate J-M.; 2008 | 3 | 1 | 2 | 6 |
| Shanab A.A.; 2011 | 4 | 1 | 2 | 7 |
| Shi H.; 2021 | 2 | 1 | 0 | 3 |
| Wigg A.J.; 2001 | 3 | 1 | 3 | 7 |

**Table S4.** Newcastle–Ottawa Scale scoring for cross–sectional studies.

| Author, Year | Summary: Selection bias (max. five stars) | Summary: Comparability (max. two stars) | Summary: Outcome (max. three stars) | Total No. Stars (Max. ten stars) |
|---|---|---|---|---|
| Fitriakusumah, Y.; 2019 | 4 | 2 | 1 | 7 |
| Lira M; 2020 | 3 | 2 | 3 | 8 |
| Mikolasevic, I.; 2021 | 3 | 2 | 3 | 8 |
| De Oliveira, J.M.; 2020 | 2 | 0 | 3 | 5 |
| Rafiei, R.; 2018 | 5 | 2 | 3 | 10 |

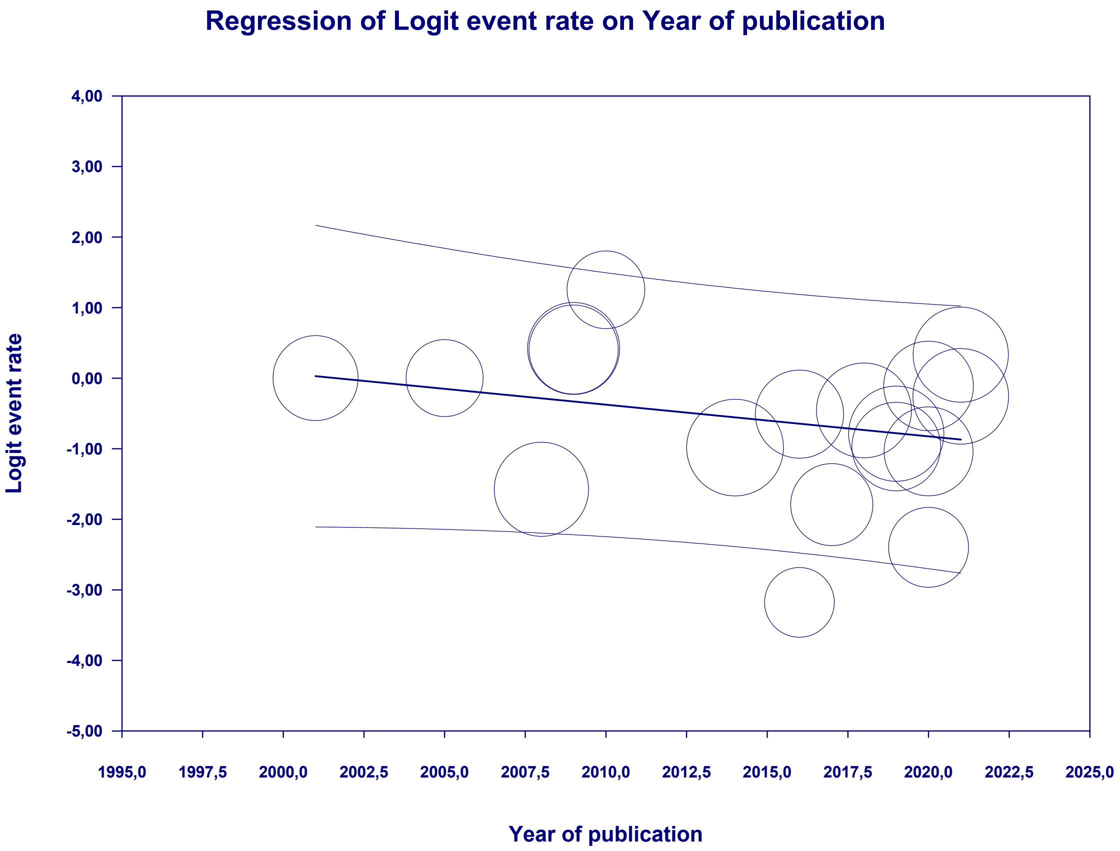


**Figure S1.** The meta-regression on log risk ratio of SIBO prevalence depending on year of publication (coefficient= -0.0450, p=0.1675)

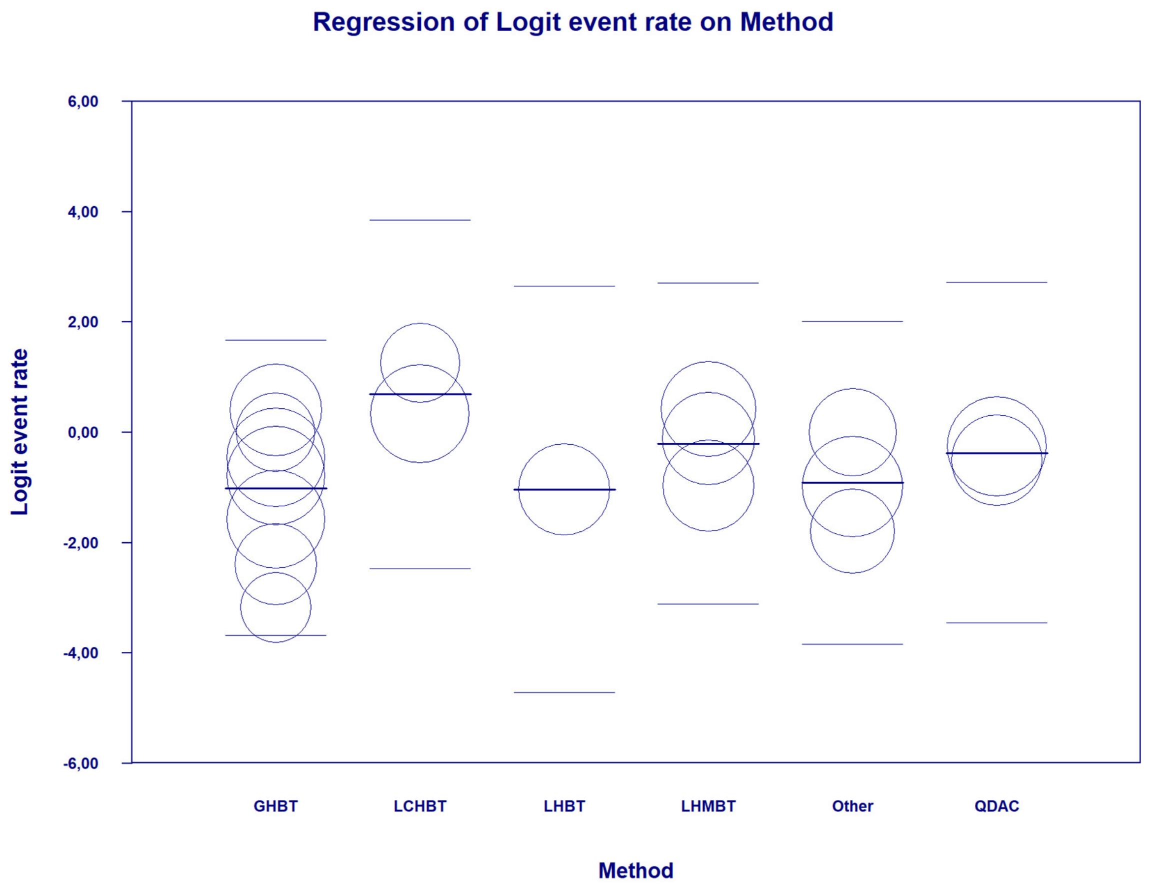


**Figure S2.** The meta-regression on log risk ratio of SIBO prevalence depending on testing method (p=0.2993); GHBT - Glucose Hydrogen Breath Test; LCHBT - Lactose Hydrogen Breath Test; LHBT - Lactulose Hydrogen Breath Test; LHMBT - Lactulose Hydrogen-Methane Breath Test; QDAC - Quantitative Duodenal Aspirate Culture
